# Microbial Epidemiology and Antimicrobial Resistance Trends in Urinary Isolates from a Tertiary Hospital in Rome, Italy: A Retrospective Study (2022–2025)

**DOI:** 10.3390/medicina62081595

**Published:** 2026-08-19

**Authors:** Fabio Ingravalle, Marco Ciotti, Giovanni Gaetti, Giustino Morlino, Giampiera Bulfone, Dorian Bardhi, Pamela Barbadoro, Francesca Pica, Stefano Di Carlo, Livio Serafinelli, Antonio Vinci, Massimo Maurici

**Affiliations:** 1 Department of Biomedicine and Prevention, University of Rome Tor Vergata, 00133 Rome, Italy; fabio.ingravalle@students.uniroma2.eu (F.I.);; 2 Local Health Authority ASL Roma 1, 00193 Rome, Italy; 3 Laboratory of Microbiology and Virology, Tor Vergata University Hospital, 00133 Rome, Italy; 4 Società Italiana di Medicina e Divulgazione (SIMED), 20054 Milan, Italy; 5 Department of Medical, Surgical Sciences and Advanced Technologies “GF Ingrassia” (DGFI), University of Catania, 95131 Catania, Italy; 6 Department Medicine, Hospital Health Management AOU delle Marche, 60126 Ancona, Italy; 7 Department of Biomedical Science and Public Health, Marche Polytechnic University, 60126 Ancona, Italy; 8 Hospital Hygiene Unit, Azienda Ospedaliero-Universitaria delle Marche, 60126 Ancona, Italy; 9 Department of Experimental Medicine, University of Rome Tor Vergata, 00133 Rome, Italy; 10 Department of Laboratory Medicine, Tor Vergata University Hospital, 00133 Rome, Italy; 11 Azienda Regionale Emergenza Sanitaria ARES 118, 00149 Roma, Italy

**Keywords:** urinary tract infections, antimicrobial resistance, hospital epidemiology

## Abstract

*Background and Objectives*: Urinary tract infections are common in clinical practice, but in hospital settings, especially among older and catheterized patients, the microbial ecology and resistance burden may differ substantially from community-acquired infections. Local surveillance is therefore essential to support appropriate empirical treatment and antimicrobial stewardship. The objective is to describe the microbiological epidemiology of urinary isolates in a tertiary hospital and evaluate temporal trends in antimicrobial resistance among the most frequently isolated microorganisms. *Materials and Methods*: This retrospective observational study analyzed microbiology laboratory data from Tor Vergata University Hospital, Rome, Italy, collected from January 2022 to June 2025. After WHONET-based deduplication using a 30-day repeat-isolate rule, 5788 deduplicated urinary isolates and 85,888 microorganism–drug associations were included. Descriptive analyses, cumulative antibiograms/antimycograms, and quarterly resistance trends were assessed using univariable and multivariable regression analyses. *Results*: The population was predominantly elderly, inpatient, and catheter-exposed. Gram-negative organisms predominated, followed by Gram-positive bacteria and fungi. The most frequent isolates were *E. coli*, *K. pneumoniae*, *E. faecalis*, and *C. albicans*. Although microorganism distribution remained broadly stable over time, resistance increased in several clinically relevant organism–drug combinations, especially among major *Enterobacterales*. *Conclusions:* In this high-complexity hospital population, urinary isolates showed a relatively stable microorganism distribution but progressive changes in susceptibility among selected urinary isolates. These findings support setting-specific microbiological surveillance and stewardship-informed empirical treatment strategies.

## 1. Introduction

Urinary tract infections (UTIs) are among the most frequently encountered infections in clinical practice and remain a major cause of healthcare utilization worldwide. Recent guidelines and consensus documents emphasize that UTIs should not be considered a homogeneous entity, as their clinical presentation, microbiological profile, and therapeutic management vary substantially according to patient characteristics, healthcare exposure, and underlying urological risk factors [1,2,3].

Within this spectrum, healthcare-associated urinary tract infections (HAUTIs), particularly catheter-associated urinary tract infections (CAUTIs), are of special relevance in hospitalized patients. Indwelling urinary catheters are a well-established risk factor for urinary infection and colonization, and catheterized patients are more likely to experience prolonged hospitalization and broader antimicrobial use. As a result, HAUTIs and CAUTIs are associated with increased morbidity, longer length of stay, and higher healthcare costs, while also posing important challenges for infection control and antimicrobial stewardship [1,4].

Although bacteria cause most urinary tract infections (UTIs), fungi—particularly *Candida* spp.—can also be clinically relevant, especially in frail or hospitalized patients with urinary devices, previous antimicrobial exposure, or other predisposing conditions. In these patients, interpreting a positive urine culture can be challenging because the finding may represent a true infection, colonization, or biofilm formation on an indwelling device [2,5,6].

The increasing prevalence of antimicrobial resistance adds further complexity to the management of UTIs. Local microbiological surveillance is therefore essential for guiding evidence-based empirical treatment, supporting antimicrobial stewardship, and identifying changes in pathogen distribution and susceptibility patterns over time. Both European and international guidelines emphasize that local epidemiological data should be incorporated into clinical decision-making for UTIs [1,2,6,7].

Within this context, laboratory-based observational studies can provide valuable real-world insights into the microbial ecology of urinary isolates. Such information is particularly important in complex healthcare settings, where advanced age, catheterization, and repeated antimicrobial exposure may shape the selection of pathogens and resistant strains. A detailed assessment of the organisms isolated and their antimicrobial susceptibility profiles can help detect emerging resistance trends, support the development of cumulative or stratified antibiograms, and improve the appropriateness of empirical treatment strategies [1,5,8].

### Study Objectives

The primary objective of this study was to describe the microbiological epidemiology of urine isolates collected during the study period, including the distribution of the main bacterial and fungal microorganisms isolated from urine cultures and their antimicrobial susceptibility profiles. Other objectives were to assess temporal trends in antimicrobial resistance among the most frequently isolated urinary microorganisms; to identify clinically relevant changes in the prevalence of specific pathogens over time and to provide local surveillance data that may support empirical therapeutic choices and antimicrobial stewardship interventions in a healthcare-associated setting.

## 2. Materials and Methods

### 2.1. Study Design

This retrospective observational epidemiological study is designed based on microbiology laboratory data, following an approach similar to that used in our previous epidemiological study of respiratory microorganisms [9]. The laboratory data included two types of samples: midstream urine and catheter urine samples. All microorganism identification tests were conducted upon medical request, along with corresponding antibiograms/antimycograms. Physicians requested microbiological testing to guide and refine empirical treatments that had already been started, whether in a healthcare facility or at the patient’s home.

### 2.2. Setting

The study was carried out at Tor Vergata University Hospital, Rome, Italy. Microbiological samples were collected from January 2022 to June 2025, covering 14 consecutive calendar quarters. The majority of samples (71.1%) were collected from hospitalized patients at the request of hospital physicians, whereas the remaining 28.9% originated from outpatients referred by their primary care physicians for culture and antimicrobial susceptibility testing. Outpatients were defined as patients without an active hospital admission at the time of specimen collection who underwent microbiological testing through the hospital outpatient diagnostic pathway. Specimens originating from nursing homes, rehabilitation facilities, home-care services, or other external healthcare facilities were not included.

Urine cultures were performed upon clinical request for suspected urinary tract infection and antimicrobial susceptibility testing. However, the laboratory database did not include information on urinary symptoms, pyuria, clinical diagnosis, quantitative culture thresholds used for clinical interpretation, or antimicrobial treatment. Therefore, positive urine cultures were analyzed as urinary isolates, and no distinction could be made between symptomatic urinary tract infection, asymptomatic bacteriuria, colonization, contamination, or catheter-associated biofilm. Between April and June 2025, the research team retrieved anonymized information from the laboratory’s electronic database.

### 2.3. Sample Selection and Study Size

**Figure 1:**
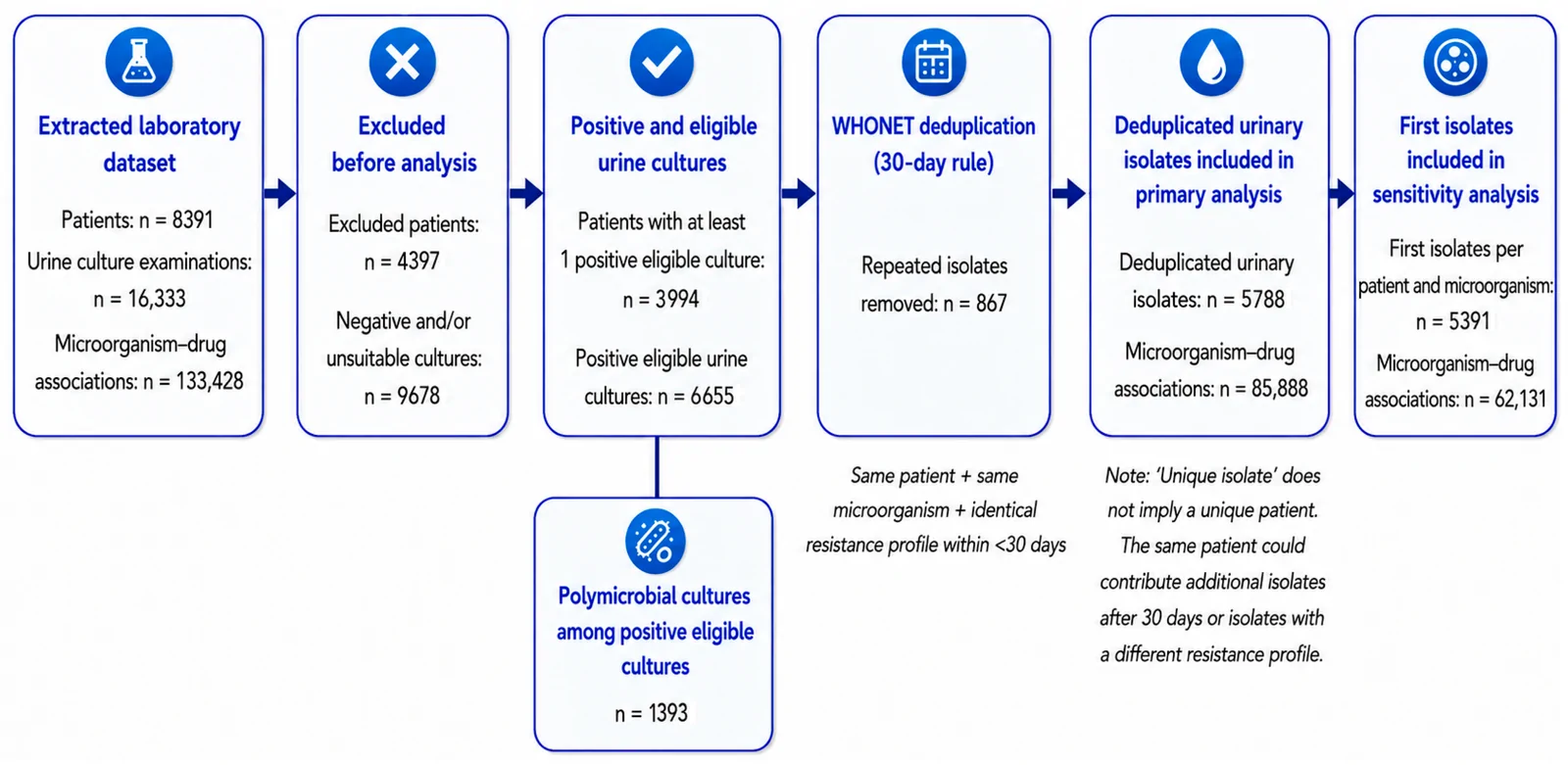
Study flow diagram.

All microbiological specimens with growth of at least one bacterial or fungal microorganism were eligible for inclusion. Cultures with no microbial growth and samples considered qualitatively or quantitatively unsuitable for analysis because of pre-analytical issues related to collection, transport, or storage were excluded according to routine laboratory acceptance criteria. Overall, 6655 positive and eligible urine cultures from 3994 patients were identified. The study selection process is summarized in Figure 1.

To reduce the influence of repeated sampling while preserving potentially relevant changes in antimicrobial susceptibility, the primary analytical dataset was generated using the WHONET repeat-isolate function. A patient-level 30-day deduplication rule was applied according to microorganism and antimicrobial resistance profile. For the same patient, isolates of the same microorganism showing an identical resistance profile within 30 days were considered repeated observations, and only the first eligible isolate within that interval was retained. Isolates obtained more than 30 days apart or showing a different antimicrobial susceptibility profile were retained.

Accordingly, a deduplicated isolate did not necessarily correspond to a unique patient because the same patient could contribute additional isolates after 30 days or isolates of the same microorganism showing a different susceptibility profile. After application of the WHONET 30-day deduplication procedure, 5788 urinary isolates, corresponding to 85,888 microorganism–drug associations, were included in the primary analysis.

To assess the potential influence of recurrent sampling, a second, more restrictive deduplication procedure was performed using the WHONET repeat-isolate function. For this sensitivity analysis, only the first isolate of each microorganism from each patient over the entire study period was retained, irrespective of subsequent sampling or changes in susceptibility profile. This procedure resulted in 5391 isolates. A single patient could still contribute one first isolate for different microorganisms.

No prior sample size calculation was performed because this was a retrospective observational study based on pre-existing laboratory data.

### 2.4. Variables

For each sample, the data reported below were extracted:

• A unique anonymous identification code assigned by the microbiology laboratory;
• Date of sample collection;
• Patient age at the time the sample was obtained;
• Patient sex;
• Ward and department of hospitalization, where applicable, or outpatient status.
• Type of urinary specimen;
• Microorganisms identified, including genus and species;
• Antimicrobial agents tested against each microorganism, including antibiotics and antifungals, with susceptibility results reported as minimum inhibitory concentration (MIC) values. Antimicrobial abbreviations are listed in the Abbreviations Table.

Healthcare setting was classified as inpatient or outpatient according to hospitalization status at the time of specimen collection. Specimen type was classified as catheter-derived or midstream urine according to the collection method recorded in the laboratory information system. For inpatients, the department variable represented the clinical department of hospitalization, whereas for outpatients it represented the administrative/diagnostic service classification recorded in the laboratory information system.

### 2.5. Data Source/Measurements

Microbiological analyses were performed using Matrix-Assisted Laser Desorption/Ionization–Time of Flight mass spectrometry (MALDI-TOF; Bruker Daltonics, Bremen, Germany), while antimicrobial susceptibility testing was conducted with the Vitek 2 system (bioMérieux, Inc., Hazelwood, MO, USA).

The resistance or susceptibility of each microorganism to the antimicrobial agents tested was determined using WHONET software (version 25.04.25), updated with the last EUCAST guidelines update [10]. Data were imported into WHONET through BacLink, a complementary tool provided by the WHO. After appropriate configuration, the software enabled the evaluation of antimicrobial resistance patterns, the generation of cumulative antibiograms, and the analyses of bacterial or fungal strains based on their phenotypic characteristics.

To determine MIC-based resistance or susceptibility, we applied the default software settings using breakpoints established by the EUCAST 2025 guidelines. These reference breakpoints were uniformly applied across all study years to ensure consistency in evaluation and to minimize potential bias in temporal trend analyses arising from changes in classification thresholds over time. This approach was based on the assumption that the latest EUCAST criteria offer enhanced diagnostic precision compared with earlier versions. Data on susceptibility rates and their interpretation were reported in accordance with the recommendations of an independent expert group on the interpretation of cumulative antibiograms and antimycograms.

For regression analyses, antimicrobial susceptibility results were dichotomized as resistant (R = 1) versus non-resistant (S or I = 0) according to EUCAST interpretation.

### 2.6. Bias and Bias Reduction

Predefined inclusion and exclusion criteria and the WHONET repeat-isolate procedures described in Section 2.3 were applied to reduce bias related to repeated sampling and overrepresentation of frequently sampled patients. Because individual patients could still contribute more than one eligible isolate to the primary dataset, patient-level cluster-robust standard errors were used in the regression analyses to account for within-patient correlation. The more restrictive WHONET-based sensitivity analysis was performed to assess the robustness of temporal trends to recurrent sampling.

Data quality was independently verified by two authors, who reviewed the electronic database and resolved discrepancies by consensus. No missing data were imputed. Antimicrobial consumption data were not available at either the patient or ward level and therefore could not be included among the covariates in the multivariable models.

This study was conducted in accordance with the STROBE reporting guidelines for observational studies.

### 2.7. Statistical Methods

Data analysis was conducted by the Department of Biomedicine and Prevention, University of Rome “Tor Vergata”. All statistical analyses were performed using Stata 19.5 SE (Standard Edition; StataCorp LLC, College Station, TX, USA). Continuous variables were summarized as mean and standard deviation (SD), and categorical variables as frequencies and percentages.

Differences across study years were assessed using one-way analysis of variance (ANOVA) for age and Pearson’s chi-squared test for categorical variables.

The observation period was divided into 14 consecutive calendar quarters, coded from 1 to 14 and modelled as a continuous temporal variable.

Temporal changes in microorganism prevalence were assessed using Pearson’s correlation coefficient and simple linear regression of quarterly standardized isolate frequencies on calendar quarter. The regression coefficient (β), its 95% confidence interval (CI), *p*-value, and R^2^ were reported; β represents the change in standardized isolate frequency associated with a one-quarter increase in time.

For each microorganism–antimicrobial combination, resistance was analyzed as a binary isolate-level outcome. Univariable logistic regression models were used to assess temporal trends, followed by multivariable models adjusted for age, sex, healthcare setting (inpatient/outpatient), specimen type, and hospital ward.

Patient-level cluster-robust standard errors were used in all regression models to account for within-patient correlation. Results are reported as odds ratios (ORs) and adjusted odds ratios (aORs), with 95% confidence intervals (CIs), representing the change in the odds of resistance associated with a one-quarter increase in time.

To account for multiple testing, *p*-values for the temporal effect were adjusted using the Benjamini–Hochberg false discovery rate (FDR) procedure, with q < 0.05 considered statistically significant. The same regression models were repeated in the sensitivity dataset defined in Section 2.3 (*n* = 5391). Models that could not be reliably estimated because of sparse data, insufficient outcome variability, or complete or quasi-complete separation were considered non-estimable and were excluded from the regression result tables.

## 3. Results

### 3.1. Patients and Clinical Specimens

Our study included 6655 urine samples collected from patients admitted to the hospital between January 2022 and June 2025. Table 1 shows the urine samples obtained over the years of the study. The mean age of the patients from whom the samples were collected was 73.5 years, with substantial stability over the years of observation. A slight prevalence of samples from female patients was also observed. Most samples came from hospitalized patients, with a proportion of in-patients appearing to progressively increase over time. The largest number of samples came from urine collected via catheter. From a microbiological point of view, mainly Gram-negative bacteria were isolated, with a significant proportion of fungal isolates, increasing over the observation period (*p*-value < 0.01). A detailed table of patient setting (inpatient or outpatient) according to specimen type and hospital or care department is provided in the Supplementary Materials Table S1.

### 3.2. Trends in Microbial Isolates in the Observation Period

Table 2 shows the main microorganisms isolated during the observation period (observations due to repeated sampling were eliminated). After the exclusion of repeated sampling from the same patients, a total of 5788 isolates were included in the analyses of this study. The most frequent microorganisms include *E. coli* and *K. pneumoniae* among Gram-negative organisms, *E. faecalis* among Gram-positive organisms, and *C. albicans* among fungi. Analysis of frequency trends shows that *E. faecalis* shows a reduction over time, as do *C. tropicalis*, *M. morganii* and *A. baumannii*. However, *C. glabrata* shows a significant increase over time. In contrast, most bacterial species isolated from urine cultures showed no significant changes over time (*p* > 0.05, low R^2^ values), indicating that their epidemiological patterns remained broadly stable.

### 3.3. Resistance Rates

Table 3 summarizes antimicrobial resistance rates over the entire study period for the main microorganism–antimicrobial combinations, whereas Table S2 provides the complete results, including the number of isolates tested and the corresponding resistant and non-resistant counts.

Among Gram-negative bacteria, *A. baumannii* showed the broadest resistance profile, with resistance exceeding 78% to most tested agents, including imipenem (92.0%), ciprofloxacin (91.5%), meropenem (90.2%), aminoglycosides, and trimethoprim/sulfamethoxazole, while no resistance to colistin was observed. *K. pneumoniae* also showed substantial resistance, particularly to ceftriaxone (89.4%), aztreonam (86.3%), ceftazidime (67.3%), ciprofloxacin (66.9%), and levofloxacin (67.4%), whereas lower resistance was observed to colistin (2.7%), amikacin (8.8%), and ceftazidime/avibactam (9.7%). *E. coli* showed high resistance to ampicillin (74.6%) and fluoroquinolones, including ciprofloxacin (51.2%), together with substantial resistance to third-generation cephalosporins; conversely, resistance to nitrofurantoin (0.3%), carbapenems (<1%), and amikacin (1.0%) remained low. *P. mirabilis* showed high resistance to ciprofloxacin (61.9%) and trimethoprim/sulfamethoxazole (59.8%), whereas *P. aeruginosa* displayed lower resistance to amikacin (4.5%), colistin (2.1%), ceftazidime/avibactam (8.0%), and ceftolozane/tazobactam (10.6%).

Among Gram-positive bacteria, *E. faecium* showed particularly high resistance to ampicillin (96.6%), ciprofloxacin (96.8%), and levofloxacin (95.5%), with resistance to vancomycin and teicoplanin of 51.0% and 53.9%, respectively. In contrast, *E. faecalis* remained largely non-resistant to glycopeptides, linezolid, ampicillin, nitrofurantoin, and tigecycline, although resistance to ciprofloxacin and levofloxacin exceeded 50%. *S. aureus* showed resistance to oxacillin in 41.4% of tested isolates, while no resistance was detected to linezolid, daptomycin, tigecycline, or trimethoprim/sulfamethoxazole. Antifungal resistance was generally low, particularly in *C. tropicalis*. *C. glabrata* showed resistance to fluconazole in 12.8% of isolates, whereas *C. albicans* showed low resistance to fluconazole (0.6%), voriconazole (0.4%), and amphotericin B (0%), although higher resistance was observed for anidulafungin among the isolates tested (26.8%).

### 3.4. Resistance Trends

Temporal trends in antimicrobial resistance were assessed using logistic regression models, with calendar quarter entered as a continuous predictor. Table 4 summarizes the associations that remained statistically significant after adjustment for potential confounders and Benjamini–Hochberg false discovery rate correction in both the primary and sensitivity analyses, while also meeting the predefined criterion for retention of observations in the adjusted primary model.

Overall, 11 microorganism–antimicrobial combinations showed robust temporal trends. Among Gram-negative bacteria, *E. coli* exhibited a significant decrease in the adjusted odds of resistance to amoxicillin/clavulanic acid (primary analysis: aOR per quarter 0.955, 95% CI 0.927–0.983; sensitivity analysis: aOR 0.951, 95% CI 0.923–0.980), amikacin (aOR 0.739, 95% CI 0.619–0.883; sensitivity: 0.761, 95% CI 0.643–0.900), and fosfomycin (aOR 0.807, 95% CI 0.704–0.926; sensitivity: 0.789, 95% CI 0.690–0.903). Conversely, *K. pneumoniae* showed increasing resistance over time to ceftazidime/avibactam, ceftolozane/tazobactam, imipenem, tobramycin, and piperacillin/tazobactam, with adjusted ORs per quarter ranging from 1.064 to 1.216 in the primary analysis and from 1.065 to 1.227 in the sensitivity analysis. A decreasing trend was observed for *K. pneumoniae* resistance to levofloxacin (aOR 0.865, 95% CI 0.796–0.939; sensitivity: 0.858, 95% CI 0.783–0.940). In addition, *P. mirabilis* showed increasing resistance to cefepime (aOR 1.161, 95% CI 1.051–1.282; sensitivity: 1.176, 95% CI 1.063–1.301).

Among fungi, *C. glabrata* showed a marked increase in resistance to amphotericin B over time, with an aOR of 1.532 per quarter (95% CI 1.231–1.906) in the primary analysis and 1.603 (95% CI 1.290–1.993) in the sensitivity analysis. All associations reported in Table 4 remained significant after FDR correction in both adjusted analyses, supporting the robustness of the observed temporal patterns to the alternative deduplication approach.

The complete results of the temporal trend analyses are reported in the Supplementary Materials: Table S3 presents the univariable primary analysis, Table S4 the multivariable primary analysis, Table S5 the univariable sensitivity analysis, and Table S6 the multivariable sensitivity analysis. Two additional associations, *C. tropicalis*–amphotericin B and *E. faecalis*–tigecycline, were FDR-significant in both adjusted primary and sensitivity analyses but were not included in Table 4 because of substantial complete-case loss; their estimates are retained in the corresponding Supplementary Tables.

## 4. Discussion

### 4.1. Key Results

This study provides a real-world overview of the microbiological epidemiology of positive urine cultures in a predominantly elderly and healthcare-exposed population, with a marked overrepresentation of inpatients and catheter-derived specimens. Among the 6655 positive eligible urine cultures, 71.1% originated from hospitalized patients and 87.2% were catheter-derived, while the mean patient age was 73.5 years. These characteristics indicate that the findings primarily reflect an elderly, healthcare-exposed, and highly catheterized population in a complex tertiary-care setting, in which positive urine cultures may include catheter-associated colonization or biofilm-related isolates. Within this context, Gram-negative microorganisms predominated, followed by Gram-positive bacteria and fungi. After application of the 30-day WHONET deduplication procedure, 5788 urinary isolates were included in the primary analytical dataset. *E. coli* and *K. pneumoniae* were the most frequently isolated Gram-negative microorganisms, *E. faecalis* the leading Gram-positive species, and *C. albicans* the most frequent fungal isolate. Overall, the major microorganism species showed relatively stable prevalence over time, whereas selected shifts were observed, including a significant increase in *C. glabrata* and reductions in *E. faecalis*, *C. tropicalis*, *M. morganii* and *A. baumannii*.

The resistance findings indicate a pattern of selective and directionally heterogeneous temporal change rather than a generalized increase in antimicrobial resistance. High overall resistance rates were observed in several clinically important microorganisms, particularly *A. baumannii*, *K. pneumoniae*, and *E. faecium*. However, high resistance prevalence did not necessarily correspond to an increasing temporal trend.

After multivariable adjustment and Benjamini–Hochberg FDR correction, 11 microorganism–antimicrobial combinations showed temporal trends that were robust in both the primary and sensitivity analyses. *E. coli* showed decreasing resistance over time to amoxicillin/clavulanic acid, amikacin, and fosfomycin. Conversely, *K. pneumoniae* showed increasing resistance to ceftazidime/avibactam, ceftolozane/tazobactam, imipenem, tobramycin, and piperacillin/tazobactam, while resistance to levofloxacin decreased. Increasing resistance was also observed for *P. mirabilis* to cefepime and for *C. glabrata* to amphotericin B.

The consistency of these associations between the primary 30-day deduplicated dataset and the more restrictive first-isolate sensitivity analysis supports their robustness to recurrent sampling. Patient-level cluster-robust standard errors further accounted for within-patient correlation. Nevertheless, these associations should be interpreted as temporal surveillance signals rather than causal effects, particularly because antimicrobial exposure and treatment data were unavailable.

### 4.2. Interpretation

Taken together, these findings indicate that the microbiological landscape captured in the present study is best interpreted as that of a high-complexity, healthcare-associated urinary culture population. This interpretation is strongly supported by the case-mix of the cohort itself. In this context, the predominance of Gram-negative organisms, the persistence of *E. coli* and *K. pneumoniae* as leading urinary isolates, and the non-negligible contribution of fungi are all biologically and clinically plausible. This is consistent with recent literature showing that catheterization, advanced age, and prolonged healthcare exposure substantially reshape urinary microbial ecology and increase the likelihood of polymicrobial and fungal findings in catheter-associated and complicated urinary conditions [11,12,13,14].

A central finding of this study is that the rank order of the main urinary microorganisms remained largely stable, whereas selected microorganism–antimicrobial combinations showed significant changes in resistance over time. Importantly, the direction of these changes was not uniform. In our cohort, *E. coli* remained the most frequent isolate and showed high overall resistance to several commonly used agents, including ciprofloxacin (51.2%), cefotaxime (39.3%), and trimethoprim/sulfamethoxazole (37.7%). However, none of these three combinations was among the robust increasing trends identified in the adjusted primary and sensitivity analyses. Instead, resistance in *E. coli* decreased over time for amoxicillin/clavulanic acid, amikacin, and fosfomycin. This distinction illustrates why cross-sectional resistance burden and temporal direction should be interpreted separately.

By comparison, the most recent ISS (Istituto Superiore di Sanità—Italian National Institute of Health) urinary surveillance report documented lower resistance in *E. coli*, including 24.7% for ciprofloxacin and 14.7% for cefotaxime. These differences should be interpreted in light of the markedly different case-mix of the present cohort, which was predominantly hospital-based and catheter-exposed.

*K. pneumoniae* represented the clearest bacterial signal of increasing resistance in the longitudinal analysis. In addition to its substantial overall resistance burden, significant FDR-robust increases were observed for ceftazidime/avibactam, ceftolozane/tazobactam, imipenem, tobramycin, and piperacillin/tazobactam in both the primary and sensitivity analyses. Of particular interest, resistance to levofloxacin decreased despite remaining high overall, again emphasizing that the absolute resistance level and its temporal trajectory provide complementary information.

The higher resistance estimates observed in our series compared with national surveillance are consistent with the interpretation that the study population represents a selected hospital cohort with substantial healthcare exposure. European surveillance data similarly indicate that resistance to third-generation cephalosporins and carbapenems is generally more pronounced in *K. pneumoniae* than in *E. coli*, with carbapenem-resistant *K. pneumoniae* remaining an important concern, particularly in several southern and eastern European countries [15,16].

The resistance patterns observed in this study are broadly consistent with recent hospital-based reports, although the estimates in our cohort appear to lie at the more severe end of the spectrum. A three-year surveillance study from a Polish teaching hospital also identified *E. coli*, *Klebsiella* spp., and *Enterococcus* spp. as the predominant urinary isolates, while showing that carbapenems, piperacillin/tazobactam, amikacin, and nitrofurantoin generally retained good activity. On that basis, the authors supported distinct empirical approaches for uncomplicated and complicated UTIs [17]. In our cohort, selected carbapenems and aminoglycosides remained active against some species, but susceptibility was less preserved in key urinary isolates, particularly *K. pneumoniae*, indicating a heavier resistance burden and limiting the applicability of standard hospital-wide antibiograms without further stratification. A recent Dutch population-based analysis reached a complementary conclusion, showing that resistance in urinary *E. coli* and *K. pneumoniae* differs across primary care, hospital outpatient, and hospital inpatient settings. Together, these findings support interpreting urinary resistance data according to the clinical setting and level of healthcare exposure, rather than applying a single empirical framework across all patient groups [18]. Together, these findings reinforce the importance of interpreting cumulative resistance data within the specific healthcare setting from which they originate.

*A. baumannii* was among the microorganisms with the highest overall resistance burden, with broad resistance across multiple antimicrobial classes, particularly carbapenems and fluoroquinolones. However, no *A. baumannii*–antimicrobial combination met the criteria for inclusion among the robust temporal trends in Table 4. Thus, its clinical relevance in this dataset derives primarily from the high overall resistance burden rather than evidence of a consistent increase during the observation period. This pattern remains consistent with wider Italian and European surveillance, in which carbapenem-resistant *Acinetobacter* spp. represent a major concern [9,15,16,19].

Another important aspect of interpretation is the fungal component of this dataset. Fungi accounted for 15.2% of all isolates, and *C. glabrata* increased significantly over time. This is much higher than the fungal yield described in many general UTIs cohorts; for example, in a recent South African retrospective series, *Candida albicans* represented only 3% of isolates [20]. By contrast, recent studies focused on hospitalized or catheter-exposed patients reported that non-albicans Candida species may increase over time, particularly in association with prolonged hospitalization, indwelling catheters, and broad-spectrum antibiotics [13,21,22]. Importantly, in the present laboratory-based dataset, recovery of *Candida* from urine should be interpreted as candiduria and does not necessarily indicate symptomatic Candida UTI. Given the high prevalence of catheterization and the absence of clinical information, some positive cultures may represent colonization or catheter-associated biofilm rather than infection. In addition, *C. glabrata* showed a robust increase in amphotericin B resistance in both adjusted analyses, despite the relatively low overall resistance proportion for this microorganism–drug combination. This finding should be interpreted cautiously given the more limited number of fungal observations, but it provides a surveillance signal that warrants continued monitoring. The observed prominence of *Candida* spp. is consistent with studies showing greater candiduria and non-albicans Candida representation among hospitalized, catheter-exposed, and medically complex patients [21,22,23].

Overall, the present findings show that a relatively stable microbial composition can coexist with meaningful and organism-specific changes in antimicrobial resistance. Surveillance should therefore consider both the prevalence of individual microorganisms and the direction of resistance trends within specific microorganism–antimicrobial combinations, rather than assuming a generalized deterioration in susceptibility.

### 4.3. Limitations

This study has some limitations inherent to its retrospective, single-center, laboratory-based design. Detailed clinical information on urinary symptoms, pyuria, comorbidities, catheter duration, antimicrobial exposure or treatment, and clinical outcomes was not available. Consequently, positive urine cultures could not be classified as symptomatic UTI, asymptomatic bacteriuria, colonization, contamination, or catheter-associated biofilm. Nevertheless, the analyses incorporated the available patient- and sample-level characteristics, including age, sex, healthcare setting, specimen type, and hospital ward, to account for relevant differences in case-mix.

Because urine cultures were obtained upon clinical request rather than through systematic population-based sampling, the findings primarily reflect the population undergoing microbiological testing at a tertiary-care hospital and may be influenced by testing practices. The total number of submitted urine cultures is reported in Figure 1; however, a conventional positivity rate could not be reliably estimated because negative cultures and unsuitable specimens were available as a combined excluded category. To improve comparability over time, EUCAST 2025 interpretative criteria were applied uniformly across all study years.

The primary 30-day WHONET deduplication procedure allowed individual patients to contribute additional isolates when predefined criteria were met. This potential overrepresentation was addressed using patient-level cluster-robust standard errors and a more restrictive sensitivity analysis retaining only the first isolate of each microorganism per patient over the entire study period. Likewise, the large number of microorganism–antimicrobial comparisons was addressed using Benjamini–Hochberg FDR correction, and the principal results were based on associations confirmed in both the primary and sensitivity analyses. Finally, although data for 2025 covered January through June only, this period comprised two complete calendar quarters and was therefore fully compatible with the quarterly framework used for temporal trend analyses.

### 4.4. Generalizability

The external validity of these findings should be considered selectively rather than broadly. Because the study population was predominantly inpatient and elderly, and largely represented by catheter urine, the present results should not be directly extrapolated to uncomplicated outpatient cystitis, primary-care UTI pathways, or younger non-catheterized populations. This limitation is not merely methodological; it is epidemiologically expected. Both comparative surveillance data and recent observational studies show that urinary resistance rates differ substantially according to setting, patient frailty, and type of care exposure. For the same reason, the higher resistance estimates seen in our cohort compared with the national ISS urinary data should be interpreted primarily as a case-mix effect, not as a contradiction of national surveillance [16]. This makes this study highly informative for similar hospital environments, but less representative of the national “average” urinary isolate population.

Another major limitation of this laboratory-based study is the lack of clinical information allowing positive urine cultures to be classified according to their clinical significance. Data on urinary symptoms, pyuria, clinical diagnosis, quantitative culture thresholds, and antimicrobial treatment were not available. Consequently, this study cannot distinguish symptomatic UTI from asymptomatic bacteriuria, colonization, contamination, or catheter-associated biofilm. In addition, antimicrobial consumption data were unavailable at both the patient and ward level and could not be included in the multivariable models; therefore, residual confounding related to antimicrobial exposure and other unmeasured clinical factors cannot be excluded.

Accordingly, the results are most generalizable to tertiary-care hospitals, internal medicine wards, geriatric units, long-stay units and other services caring for older, device-exposed, and highly healthcare-exposed patients. In these contexts, the combination of persistent *Enterobacterales* dominance, increasing resistance in *K. pneumoniae*, poor susceptibility in selected non-fermenters, and a measurable burden of candiduria is highly plausible and relevant to local microbiological surveillance and antimicrobial stewardship [12,13,22]. Hospital-based surveillance studies from Poland and other inpatient cohorts similarly suggest that local urinary epidemiology in complex care settings can differ materially from community-oriented assumptions and should therefore inform setting-specific empirical treatment strategies [17].

By contrast, these findings should not be directly extrapolated to community settings, as this would likely overestimate the burden of resistance and underestimate the continued effectiveness of first-line agents in lower-risk patients. The main generalizable implication is therefore that urinary microbiology and resistance patterns in frail, catheterized hospital patients may differ markedly from those observed in community populations.

## 5. Conclusions

This study shows that, in a predominantly elderly, hospitalized, and highly catheter-exposed population, the distribution of urinary isolates remained broadly stable over time, while selected microorganism–antimicrobial combinations exhibited significant and directionally heterogeneous changes in resistance. Robust increases were observed particularly among selected *K. pneumoniae*, *P. mirabilis*, and *C. glabrata* combinations, whereas resistance decreased for selected *E. coli* and *K. pneumoniae* combinations. The consistency of these findings across the primary and sensitivity analyses supports their value as local antimicrobial resistance surveillance signals. However, because positive urine cultures could not be classified according to their clinical significance, and antimicrobial exposure data were unavailable, these findings should be interpreted within the specific hospital case-mix and should not alone determine empirical treatment choices. Setting-specific microbiological surveillance, integrated with clinical and antimicrobial-use information, may support antimicrobial stewardship and the periodic reassessment of local therapeutic strategies.

## Figures and Tables

**Table 1:** Annual distribution of positive eligible urine cultures according to patient characteristics, healthcare setting of origin, specimen type, and microbial isolate category.

Variables	2022	2023	2024	2025 ^α^	Total	*p*-Value
Age	73.85 ± 13.47	73.17 ± 13.78	74.1 ± 13.3	71.98 ± 15.11	73.5 ± 13.72	0.78
Male	43.04% (*n* = 853)	43.48% (*n* = 900)	44.26% (*n* = 825)	47.09% (*n* = 348)	43.97% (*n* = 2926)	0.10
Female	56.96% (*n* = 1129)	56.52% (*n* = 1170)	55.74% (*n* = 1039)	52.91% (*n* = 391)	56.03% (*n* = 3729)	
Inpatient	67.36% (*n* = 1335)	71.01% (*n* = 1470)	73.93% (*n* = 1378)	74.15% (*n* = 548)	71.09% (*n* = 4731)	0.23
Outpatient	32.64% (*n* = 647)	28.99% (*n* = 600)	26.07% (*n* = 486)	25.85% (*n* = 191)	28.91% (*n* = 1924)	
Urine, catheter	88.7% (*n* = 1758)	87% (*n* = 1801)	87.02% (*n* = 1622)	84.44% (*n* = 624)	87.23% (*n* = 5805)	0.12
Urine, midstream	11.3% (*n* = 224)	13% (*n* = 269)	12.98% (*n* = 242)	15.56% (*n* = 115)	12.77% (*n* = 850)	
Gram−	62.97% (*n* = 1248)	66.18% (*n* = 1370)	64.81% (*n* = 1208)	61.43% (*n* = 454)	64.31% (*n* = 4280)	<0.01
Gram+	22.65% (*n* = 449)	17.44% (*n* = 361)	21.57% (*n* = 402)	20.16% (*n* = 149)	20.45% (*n* = 1361)	
Fungi	14.38% (*n* = 285)	16.38% (*n* = 339)	13.63% (*n* = 254)	18.4% (*n* = 136)	15.24% (*n* = 1014)	
Total positive eligible urine cultures	*n* = 1982	*n* = 2070	*n* = 1864	*n* = 739	*n* = 6655	-

^α^: For the year 2025, the months from January to June were selected; data are presented as mean ± standard deviation (SD) for age and as n (%) for categorical variables. Frequencies for healthcare setting (inpatient/outpatient) indicate the number of positive eligible urine cultures originating from each setting and should not be interpreted as numbers of unique patients. Specimen-type frequencies indicate the number of cultures obtained from catheter-derived or midstream urine. Microbial categories indicate the type of microorganism isolated from each sample.

**Table 2:** Distribution of isolated microorganisms and temporal trends in their frequency by calendar quarter.

Microorganism	Group	Number of Isolates	Pearson Coef. ^γ^	β Coef. ^δ^	95% CI β Coef. ^ε^	*p*-Value	R^2^
*E. coli*	gram−	1546	−0.269	−0.141	−0.306–0.023	0.09	0.072
*K. pneumoniae*	gram−	821	−0.214	−0.074	−0.182–0.035	0.18	0.046
*E. faecalis*	gram+	688	−0.41	−0.184	−0.316–−0.051	<0.01	0.168
*C. albicans*	fungi	468	−0.075	−0.021	−0.111–0.069	0.639	0.006
*E. faecium*	gram+	465	−0.007	−0.002	−0.111–0.106	0.96	0
*P. aeruginosa*	gram−	444	−0.002	−0.001	−0.105–0.103	0.989	0
*P. mirabilis*	gram−	389	−0.089	−0.019	−0.086–0.049	0.58	0.008
*C. glabrata*	fungi	170	0.534	0.11	0.054–0.167	<0.001	0.285
*C. tropicalis*	fungi	156	−0.415	−0.079	−0.136–−0.023	<0.01	0.172
*E. cloacae*	gram−	71	−0.12	−0.013	−0.049–0.022	0.455	0.014
*C. koseri*	gram−	67	0.201	0.024	−0.014–0.061	0.207	0.041
*M. morganii*	gram−	66	−0.462	−0.043	−0.07–−0.016	<0.01	0.214
*A. baumannii*	gram−	59	−0.447	−0.066	−0.108–−0.023	<0.01	0.2
*S. aureus*	gram+	56	−0.192	−0.017	−0.044–0.011	0.229	0.037
Others Gram−	gram−	246	n.a.	n.a.	n.a.	n.a.	n.a.
Others Gram+	gram+	20	n.a.	n.a.	n.a.	n.a.	n.a.
Others Fungi	fungi	56	n.a.	n.a.	n.a.	n.a.	n.a.
Total	n.a.	5788	n.a.	n.a.	n.a.	n.a.	n.a.

^γ^: Pearson coefficient is a statistical index that measures the strength and direction of the linear relationship between two variables (positive value for increase, negative value for decrease); ^δ^: regression coefficient is a statistical index that measures how the dependent variable (number of standardized isolates) changes when the independent variable (quarter—time) varies by one unit; ^ε^: 95% confidence interval of the β regression coefficient (inf, sup).

**Table 3:** Antimicrobial resistance of urinary isolates included in the primary 30-day deduplicated analyses for Gram- bacteria (Panel A), for Gram+ bacteria (Panel B) and for fungi (Panel C).

**(A)**									
**Antimicrobial**	***E. coli***	***K. pneumoniae***	***P. aeruginosa***	***P. mirabilis***	***E. cloacae***	***C. koseri***	***M. morganii***	***K. aerogenes***	***A. baumannii***
Amikacin	15/1525 (1.0%)	71/810 (8.8%)	20/443 (4.5%)	46/338 (13.6%)	1/72 (1.4%)	0/67 (0.0%)	1/66 (1.5%)	0/53 (0.0%)	48/61 (78.7%)
Amoxicillin/Clavulanic acid	146/1552 (9.4%)	234/713 (32.8%)	Not reported	34/363 (9.4%)	26/70 (37.1%)	1/66 (1.5%)	10/62 (16.1%)	21/52 (40.4%)	Not reported
Ampicillin	91/122 (74.6%)	Not reported	Not reported	70/101 (69.3%)	Not reported	Not reported	Not reported	Not reported	Not reported
Ampicillin/Sulbactam	Not reported	Not reported	Not reported	Not reported	Not reported	Not reported	Not reported	Not reported	Not reported
Aztreonam	Not reported	113/131 (86.3%)	31/195 (15.9%)	Not reported	Not reported	Not reported	Not reported	Not reported	Not reported
Cefepime	413/1439 (28.7%)	528/826 (63.9%)	87/452 (19.2%)	45/288 (15.6%)	16/70 (22.9%)	3/62 (4.8%)	0/59 (0.0%)	3/53 (5.7%)	Not reported
Cefiderocol	Not reported	Not reported	Not reported	Not reported	Not reported	Not reported	Not reported	Not reported	Not reported
Cefotaxime	540/1375 (39.3%)	282/480 (58.8%)	Not reported	113/251 (45.0%)	30/62 (48.4%)	6/59 (10.2%)	5/54 (9.3%)	26/46 (56.5%)	Not reported
Cefpodoxime	53/122 (43.4%)	Not reported	Not reported	46/101 (45.5%)	Not reported	Not reported	Not reported	Not reported	Not reported
Ceftazidime	543/1563 (34.7%)	518/770 (67.3%)	106/450 (23.6%)	137/389 (35.2%)	42/72 (58.3%)	6/66 (9.1%)	7/66 (10.6%)	31/53 (58.5%)	Not reported
Ceftazidime/Avibactam	9/147 (6.1%)	61/630 (9.7%)	35/435 (8.0%)	5/149 (3.4%)	3/35 (8.6%)	Not reported	0/41 (0.0%)	Not reported	Not reported
Ceftibuten	Not reported	Not reported	Not reported	Not reported	Not reported	Not reported	Not reported	Not reported	Not reported
Ceftolozane/Tazobactam	29/148 (19.6%)	372/629 (59.1%)	47/442 (10.6%)	21/149 (14.1%)	22/36 (61.1%)	Not reported	0/40 (0.0%)	Not reported	Not reported
Ceftriaxone	65/155 (41.9%)	168/188 (89.4%)	Not reported	48/108 (44.4%)	Not reported	Not reported	Not reported	Not reported	Not reported
Cefuroxime	Not reported	Not reported	Not reported	Not reported	Not reported	Not reported	Not reported	Not reported	Not reported
Ciprofloxacin	729/1424 (51.2%)	486/726 (66.9%)	93/450 (20.7%)	240/388 (61.9%)	13/67 (19.4%)	3/64 (4.7%)	14/66 (21.2%)	3/49 (6.1%)	54/59 (91.5%)
Colistin	6/565 (1.1%)	20/746 (2.7%)	8/383 (2.1%)	156/157 (99.4%)	2/35 (5.7%)	0/44 (0.0%)	43/44 (97.7%)	0/40 (0.0%)	0/61 (0.0%)
Doripenem	Not reported	Not reported	Not reported	Not reported	Not reported	Not reported	Not reported	Not reported	Not reported
Ertapenem	23/1458 (1.6%)	168/397 (42.3%)	Not reported	3/251 (1.2%)	12/40 (30.0%)	0/44 (0.0%)	Not reported	7/36 (19.4%)	Not reported
Fosfomycin	18/325 (5.5%)	Not reported	Not reported	Not reported	Not reported	Not reported	Not reported	Not reported	Not reported
Gentamicin	288/1562 (18.4%)	285/769 (37.1%)	Not reported	136/366 (37.2%)	6/72 (8.3%)	0/66 (0.0%)	5/62 (8.1%)	1/53 (1.9%)	47/57 (82.5%)
Imipenem	9/985 (0.9%)	154/492 (31.3%)	80/442 (18.1%)	21/272 (7.7%)	1/46 (2.2%)	0/40 (0.0%)	1/56 (1.8%)	Not reported	46/50 (92.0%)
Levofloxacin	247/585 (42.2%)	242/359 (67.4%)	24/58 (41.4%)	65/119 (54.6%)	Not reported	Not reported	Not reported	Not reported	Not reported
Meropenem	14/1570 (0.9%)	306/839 (36.5%)	56/452 (12.4%)	2/392 (0.5%)	1/73 (1.4%)	0/67 (0.0%)	0/66 (0.0%)	1/53 (1.9%)	55/61 (90.2%)
Meropenem/Vaborbactam	Not reported	19/135 (14.1%)	Not reported	Not reported	Not reported	Not reported	Not reported	Not reported	Not reported
Moxifloxacin	Not reported	Not reported	Not reported	Not reported	Not reported	Not reported	Not reported	Not reported	Not reported
Nitrofurantoin	3/976 (0.3%)	Not reported	Not reported	Not reported	Not reported	Not reported	Not reported	Not reported	Not reported
Piperacillin	Not reported	52/57 (91.2%)	21/42 (50.0%)	Not reported	Not reported	Not reported	Not reported	Not reported	Not reported
Piperacillin/Tazobactam	184/1408 (13.1%)	476/787 (60.5%)	138/451 (30.6%)	11/288 (3.8%)	33/66 (50.0%)	3/59 (5.1%)	1/58 (1.7%)	30/52 (57.7%)	Not reported
Tigecycline	2/54 (3.7%)	Not reported	Not reported	Not reported	Not reported	Not reported	Not reported	Not reported	Not reported
Tobramycin	31/102 (30.4%)	188/398 (47.2%)	48/438 (11.0%)	49/138 (35.5%)	4/31 (12.9%)	Not reported	3/41 (7.3%)	Not reported	42/50 (84.0%)
Trimethoprim/Sulfamethoxazole	585/1551 (37.7%)	423/722 (58.6%)	Not reported	217/363 (59.8%)	9/71 (12.7%)	2/65 (3.1%)	17/62 (27.4%)	0/52 (0.0%)	48/57 (84.2%)
(**B**)									
**Antimicrobial**	***E. faecalis***			***E. faecium***			***S. aureus***		
Ampicillin	6/694 (0.9%)			454/470 (96.6%)			Not reported		
Cefoxitin	Not reported			Not reported			Not reported		
Ceftaroline	Not reported			Not reported			0/47 (0.0%)		
Ciprofloxacin	349/652 (53.5%)			420/434 (96.8%)			Not reported		
Clindamycin	Not reported			Not reported			5/58 (8.6%)		
Daptomycin	Not reported			Not reported			0/58 (0.0%)		
Erythromycin	Not reported			Not reported			33/58 (56.9%)		
Fusidic acid	Not reported			Not reported			4/58 (6.9%)		
Gentamicin	Not reported			Not reported			5/58 (8.6%)		
Imipenem	6/693 (0.9%)			Not reported			Not reported		
Levofloxacin	356/653 (54.5%)			426/446 (95.5%)			32/58 (55.2%)		
Linezolid	3/695 (0.4%)			17/472 (3.6%)			0/58 (0.0%)		
Nitrofurantoin	2/648 (0.3%)			Not reported			Not reported		
Oxacillin	Not reported			Not reported			24/58 (41.4%)		
Penicillin G	Not reported			Not reported			46/58 (79.3%)		
Quinupristin/Dalfopristin	Not reported			79/458 (17.2%)			Not reported		
Rifampin	Not reported			Not reported			2/58 (3.4%)		
Streptomycin-High	Not reported			Not reported			Not reported		
Teicoplanin	14/695 (2.0%)			254/471 (53.9%)			1/58 (1.7%)		
Tetracycline	Not reported			Not reported			2/58 (3.4%)		
Tigecycline	1/693 (0.1%)			20/471 (4.2%)			0/58 (0.0%)		
Trimethoprim/Sulfamethoxazole	Not reported			Not reported			0/58 (0.0%)		
Vancomycin	11/693 (1.6%)			240/471 (51.0%)			1/58 (1.7%)		
**(C)**									
**Antimicrobial**	***C. albicans***			***C. glabrata***			***C. tropicalis***		
Amphotericin B	0/471 (0.0%)			10/172 (5.8%)			1/160 (0.6%)		
Anidulafungin	11/41 (26.8%)			9/172 (5.2%)			Not reported		
Fluconazole	3/473 (0.6%)			22/172 (12.8%)			3/160 (1.9%)		
Itraconazole	5/42 (11.9%)			Not reported			Not reported		
Micafungin	2/33 (6.1%)			1/148 (0.7%)			Not reported		
Posaconazole	Not reported			Not reported			Not reported		
Voriconazole	1/276 (0.4%)			Not reported			1/155 (0.6%)		

Data are reported as *n*/N (%), where *n* is the number of resistant isolates, and N is the number of isolates tested for each microorganism–antimicrobial combination. Estimates based on fewer than 30 tested isolates are reported as “Not reported”. Complete results are provided in Supplementary Table S2.

**Table 4:** Adjusted temporal trends in antimicrobial resistance in the primary and sensitivity analyses.

Microorganism	Antimicrobial	Adjusted Primary Analysis				Adjusted Sensitivity Analysis				Resistance Trend over Time
		N	aOR per Quarter (95% CI)	*p*-Value	BH-FDR q-Value	N	aOR per Quarter (95% CI)	*p*-Value	BH-FDR q-Value	
*C. glabrata*	Amphotericin B	166	1.532 (1.231–1.906)	0.0001	0.0048	156	1.603 (1.290–1.993)	<0.0001	0.0008	↑ Increasing
*E. coli*	Amoxicillin/clavulanic acid	1538	0.955 (0.927–0.983)	0.0018	0.0298	1401	0.951 (0.923–0.980)	0.0010	0.0167	↓ Decreasing
*E. coli*	Amikacin	1411	0.739 (0.619–0.883)	0.0008	0.0176	1286	0.761 (0.643–0.900)	0.0014	0.0193	↓ Decreasing
*E. coli*	Fosfomycin	253	0.807 (0.704–0.926)	0.0023	0.0331	221	0.789 (0.690–0.903)	0.0006	0.0131	↓ Decreasing
*K. pneumoniae*	Ceftazidime-avibactam	626	1.145 (1.052–1.246)	0.0017	0.0298	511	1.194 (1.070–1.331)	0.0015	0.0193	↑ Increasing
*K. pneumoniae*	Ceftolozane-tazobactam	625	1.187 (1.128–1.249)	<0.0001	<0.0001	510	1.189 (1.128–1.253)	<0.0001	<0.0001	↑ Increasing
*K. pneumoniae*	Imipenem	490	1.216 (1.143–1.295)	<0.0001	<0.0001	411	1.227 (1.145–1.314)	<0.0001	<0.0001	↑ Increasing
*K. pneumoniae*	Levofloxacin	353	0.865 (0.796–0.939)	0.0006	0.0141	296	0.858 (0.783–0.940)	0.0011	0.0167	↓ Decreasing
*K. pneumoniae*	Tobramycin	391	1.205 (1.135–1.280)	<0.0001	<0.0001	324	1.222 (1.145–1.305)	<0.0001	<0.0001	↑ Increasing
*K. pneumoniae*	Piperacillin-tazobactam	784	1.064 (1.020–1.109)	0.0042	0.0478	656	1.065 (1.021–1.111)	0.0037	0.0351	↑ Increasing
*P. mirabilis*	Cefepime	275	1.161 (1.051–1.282)	0.0033	0.0436	254	1.176 (1.063–1.301)	0.0017	0.0203	↑ Increasing

aOR, adjusted odds ratio for a one-quarter increase in calendar time; CI, confidence interval; BH-FDR, Benjamini–Hochberg false discovery rate. Primary analysis: WHONET 30-day deduplicated dataset. Sensitivity analysis: only the first isolate of each microorganism from each patient over the entire study period was retained, irrespective of subsequent sampling or changes in susceptibility profile. Multivariable models were adjusted for age, sex, hospital ward, healthcare setting, and specimen type, with patient-level cluster-robust standard errors. Two additional adjusted associations were BH-FDR significant in both primary and sensitivity analyses but were not included because of marked complete-case loss: Candida tropicalis–amphotericin B (20/157; 12.7% retained) and Enterococcus faecalis–tigecycline (86/688; 12.5% retained). These results are reported in Supplementary Tables S4 and S6.

## Data Availability

Individual-level data supporting the findings of this study cannot be made publicly available because of privacy and data protection requirements under the General Data Protection Regulation (GDPR; EU Regulation 2016/679). However, the aggregated and anonymized data used in the analyses are provided in the Supplementary Materials accompanying this article.

## Supplementary Materials

The following supporting information can be downloaded at: https://www.mdpi.com/article/10.3390/medicina62081595/s1, Table S1: Distribution of positive eligible urine cultures by healthcare setting, specimen type, and ward of origin; Table S2: Detailed antimicrobial resistance and susceptibility results by microorganism–antimicrobial combination; Table S3: Univariable logistic regression analysis of temporal antimicrobial resistance trends in the primary 30-day deduplicated dataset; Table S4: Multivariable logistic regression analysis of temporal antimicrobial resistance trends in the primary 30-day deduplicated dataset; Table S5: Univariable logistic regression analysis of temporal antimicrobial resistance trends in the first-isolate sensitivity dataset; Table S6: Multivariable logistic regression analysis of temporal antimicrobial resistance trends in the first-isolate sensitivity dataset.

## Abbreviations

The following abbreviations are used in this manuscript:

aOR	Adjusted Odds Ratio
ANOVA	Analysis of Variance
BH-FDR	Benjamini–Hochberg False Discovery Rate
CAUTIs	Catheter-associated urinary tract infections
CI	Confidence interval
Coef.	Coefficient
ECDC	European Centre for Disease Prevention and Control
EUCAST	European Committee on Antimicrobial Susceptibility Testing
FDR	False Discovery Rate
GDPR	General Data Protection Regulation
Gram−	Gram-negative
Gram+	Gram-positive
HAUTIs	Healthcare-associated urinary tract infections
ICH-GCP	International Council for Harmonisation—Good Clinical Practice
ID	Identifier
ISS	Istituto Superiore di Sanità—Italian National Institute of Health
MALDI-TOF	Matrix-Assisted Laser Desorption/Ionization–Time of Flight
MIC	Minimum inhibitory concentration
*n*	Number of observations or subjects
N/A; n.a.	Not applicable
OR	Odds Ratio
R^2^	Coefficient of determination
SD	Standard Deviation
spp.	Species, plural form
STROBE	Strengthening the Reporting of Observational Studies in Epidemiology
UTIs	Urinary tract infections
WHO	World Health Organization
WHONET	WHO software for the surveillance and analysis of antimicrobial resistance
AMB	Amphotericin B
AMC	Amoxicillin/clavulanic acid
AMK	Amikacin
AMP	Ampicillin
AMX	Amoxicillin
ANI	Anidulafungin
ATM	Aztreonam
AZL	Azlocillin
BPR	Ceftobiprole
CAS	Caspofungin
CAZ	Ceftazidime
CEP	Cephalothin
CFM	Cefixime
CHL	Chloramphenicol
CIP	Ciprofloxacin
CLI	Clindamycin
COL	Colistin
CPD	Cefpodoxime
CPT	Ceftaroline
CRO	Ceftriaxone
CTB	Ceftibuten
CTX	Cefotaxime
CXM	Cefuroxime
CZA	Ceftazidime/avibactam
CZT	Ceftolozane/tazobactam
DAL	Dalbavancin
DAP	Daptomycin
DOR	Doripenem
DOX	Doxycycline
ERY	Erythromycin
ETP	Ertapenem
FCT	5-Fluorocytosine
FDC	Cefiderocol
FEP	Cefepime
FLU	Fluconazole
FOS	Fosfomycin
FOX	Cefoxitin
FUS	Fusidic acid
GEN	Gentamicin
IPM	Imipenem
ITR	Itraconazole
LNZ	Linezolid
LVX	Levofloxacin
MEM	Meropenem
MEV	Meropenem/vaborbactam
MFX	Moxifloxacin
MIF	Micafungin
MNO	Minocycline
MUP	Mupirocin
NIT	Nitrofurantoin
OXA	Oxacillin
PEN	Penicillin G
PIP	Piperacillin
POS	Posaconazole
QDA	Quinupristin/dalfopristin
RIF	Rifampin
SAM	Ampicillin/sulbactam
STH	High-level streptomycin
SXT	Trimethoprim/sulfamethoxazole
TCY	Tetracycline
TEC	Teicoplanin
TGC	Tigecycline
TOB	Tobramycin
TZD	Tedizolid
TZP	Piperacillin/tazobactam
VAN	Vancomycin
VOR	Voriconazole
